# Association of novel visceral obesity indices with 10-year risk of major cardiovascular events in patients with type 2 diabetes mellitus

**DOI:** 10.1186/s42506-025-00188-w

**Published:** 2025-05-30

**Authors:** Mirella Y. Tawfik, Samar F. Mohamed, Sally F. Elotla

**Affiliations:** 1 Department of Public Health, Occupational and Environmental Medicine, Faculty of Medicine, Suez Canal University, Ismailia, Egypt; 2 Department of Family Medicine, Faculty of Medicine, Suez Canal University, Ismailia, Egypt

**Keywords:** Cardiovascular disease, Type 2 diabetes mellitus, Novel visceral obesity indices, Lipid accumulation product, Visceral adiposity index, Metabolic score for visceral fat

## Abstract

**Background:**

Cardiovascular disease (CVD) is a leading cause of mortality among individuals with type 2 diabetes mellitus (T2DM), with visceral adiposity being a key contributor to increased CVD risk. Novel visceral obesity indices (NVOI), including the lipid accumulation product (LAP), visceral adiposity index (VAI), and metabolic score of visceral fat (METS-VF), offer improved visceral adipose tissue assessment and may enhance CVD risk prediction. This study aimed to evaluate the association of these indices with 10-year CVD risk and their predictive performance in adults with T2DM.

**Methods:**

A cross-sectional study was conducted in the diabetes outpatient clinic and family medicine units of Suez Canal University in Ismailia, Egypt over 15 months starting in February 2023. A total of 397 randomly selected patients with T2DM participated. A structured interview questionnaire was used to collect demographics, medical, family, and lifestyle-related data. Clinical data such as blood pressure, body mass index (BMI), waist circumference (WC), and laboratory data such as fasting blood glucose (FBG) and lipid profile were obtained. NVOIs were calculated using standardized equations, and 10-year CVD risk was determined using the 2019 WHO/ISH CVD risk-laboratory-based chart. Logistic regression was used to assess the associations between NVOIs and high CVD risk, while receiver operating characteristic (ROC) curve analysis was used to evaluate its predictive accuracy.

**Results:**

High CVD risk (≥ 20% 10-year risk) was identified in 40.5% of participants and was significantly associated with higher LAP, VAI, and METS-VF levels (*p* < 0.001). VAI was associated with 3.18 times higher odds of having a high 10-year CVD risk (95% CI 1.61–6.26, *p* < 0.001) in males and 4.16 (95% CI 1.26–13.68, *p* = 0.019) in females. METS-VF had the highest predictive ability, with an adjusted odds ratio (aOR) of 7.39 (95% CI 1.03–52.85, *p* = 0.046) in males and 7.80 (95% CI 1.53–39.92, *p* = 0.014) in females while, LAP showed no significant association. The area under the curve (AUC) values indicated acceptable to excellent predictive accuracy for all indices, with METS-VF and VAI generally outperforming LAP. VAI performs best in males and METS-VF in females. Sensitivity ranged from 63.92 to 87.5%, while specificity varied between 73.79% and 94.51%. Positive predictive values (PPVs) were higher in males (77–92.5%), whereas negative predictive values (NPVs) were higher in females (88.9–93%).

**Conclusions:**

High CVD risk was significantly associated with elevated VAI, METS-VF, and LAP; however, only VAI and METS-VF emerged as independent predictors. These indices demonstrated the highest predictive accuracy, reinforcing their clinical relevance. Given their superior discriminative ability, incorporating VAI and METS-VF into routine assessments could enhance CVD risk prediction in adults with T2DM, allowing for earlier intervention and better management strategies.

## Introduction

Cardiovascular disease (CVD) constitutes a significant health challenge for individuals with type 2 diabetes mellitus (T2DM), affecting approximately 32% of this population and contributing significantly to global mortality [1]. Concurrently, the prevalence of obesity and T2DM has been rising at an alarming rate, particularly among younger populations, where T2DM now accounts for 25–45% of all diabetes cases in youth [2]. Given the elevated CVD risk in individuals with T2DM compared to non-diabetics, regular risk assessment is essential for estimating the 10-year probability of major cardiovascular events and optimizing prevention strategies [3–5]. In this context, absolute CVD risk assessment, which estimates the likelihood of a major cardiovascular event (e.g., myocardial infarction or stroke) within 10 years, has become an essential tool in clinical practice.

While the World Health Organization/International Society of Hypertension 2019 cardiovascular risk prediction charts (WHO/ISH- 2019) provide valuable tools for risk assessment [6], they do not address modifiable factors like visceral adipose tissue (VAT), a well-established CVD risk factor in T2DM [7]. However, the gold-standard methods for assessing VAT, such as dual-energy X-ray absorptiometry and magnetic resonance imaging, are often impractical for routine clinical use due to equipment and technical constraints, highlighting the need for surrogate markers [8].

Anthropometric measures, such as waist circumference (WC), waist-to-height ratio (WhtR), and waist-to-hip ratio, while frequently used as proxies for VAT [9], have limitations in differentiating between subcutaneous and visceral adipose tissue [10]. To address these limitations, researchers have focused on developing novel visceral obesity indices (NVOIs) that combine anthropometric and laboratory-based parameters. These indices are particularly relevant for T2DM patients, as insulin resistance often occurs when fat accumulates in intra-abdominal depots and is associated with a constellation of CVD risk factors, in what is known as the metabolic syndrome [11]. The lipid accumulation product (LAP), visceral adiposity index (VAI), and metabolic score of visceral fat (METS-VF) represent promising examples of these indices.

The lipid accumulation product (LAP), derived from a combination of waist circumference (WC) and triglyceride (TG) levels, has been widely recognized as a sensitive and specific marker for differentiating visceral fat [12]. By incorporating both anatomical distribution and physiological impact [13], LAP offers a practical alternative to gold-standard VAT measurement methods. Previous research has highlighted LAP’s strong association with visceral obesity and its potential utility as a predictive marker for CVD risk in individuals with T2DM [12].

The visceral adiposity index represents another novel surrogate marker of VAT accumulation and dysfunction [14, 15]. It incorporates body mass index (BMI), WC, TG, and high-density lipoprotein cholesterol (HDL-C). Studies have demonstrated a strong correlation between VAI and VAT, emphasizing its utility as a proxy for visceral fat [14]. Moreover, VAI has been linked to an increased risk of coronary heart disease, highlighting its potential as a cardiovascular risk predictor [16]. The metabolic score for visceral fat, a comprehensive index incorporating BMI, fasting blood glucose (FBG), TG, HDL-C, WhtR, age, and an estimate of insulin resistance (IR) [8], has demonstrated strong correlations with VAT levels measured by both dual-energy X-ray absorptiometry and magnetic resonance imaging, validating its accuracy as a VAT estimator [8]. Furthermore, research has linked METS-VF to an increased risk of CVD events, suggesting its potential as a predictive index for CVD risk [17].

Although previous studies have explored the relationship between visceral obesity and CVD risk, research on the predictive ability of NVOIs in patients with T2DM remains limited. Most existing studies have either focused on the general population or assessed these indices in relation to established CVD events rather than long-term risk prediction [17, 18]. Notably, while the LAP and VAI have been investigated in some diabetic populations, their ability to predict 10-year CVD risk in individuals without prior cardiovascular events remains largely unexplored [19, 20]. Moreover, the role of METS-VF in predicting CVD risk, particularly in T2DM patients, has not been previously examined. Given the high prevalence of obesity [21] and increased CVD risk in T2DM patients [3], identifying reliable surrogate markers for visceral adiposity that can enhance risk stratification is crucial. The standard WHO/ISH CVD risk prediction charts do not account for VAT, a well-established contributor to cardiometabolic disease [7]. Understanding how these indices relate to future CVD risk could improve clinical decision-making by providing additional tools for risk stratification, particularly in resource-limited settings. This study seeks to bridge this gap by evaluating the association of LAP, VAI, and METS-VF with 10-year CVD risk and assessing their predictive performance.

## Methods

### Study design, setting and participants

This cross-sectional study was conducted in the diabetes outpatient clinic of Suez Canal University Hospital and family medicine units of Suez Canal University in Ismailia, Egypt, from February 2023 to April 2024. Patients aged 40 years or older with type 2 diabetes mellitus who attended these settings for follow-up were recruited. Exclusion criteria included hypothyroidism, Cushing’s disease, polycystic ovarian syndrome, congestive heart failure, chronic liver disease, chronic kidney disease, cancer, and current use of statins. Additionally, patients taking birth control pills, antipsychotics, antidepressants, epilepsy drugs, or beta-blockers, as well as those with a history of cardiovascular events (defined as being diagnosed with or treated for myocardial infarction or stroke), were excluded.

### Sample size and sampling procedure

**Fig. 1:**
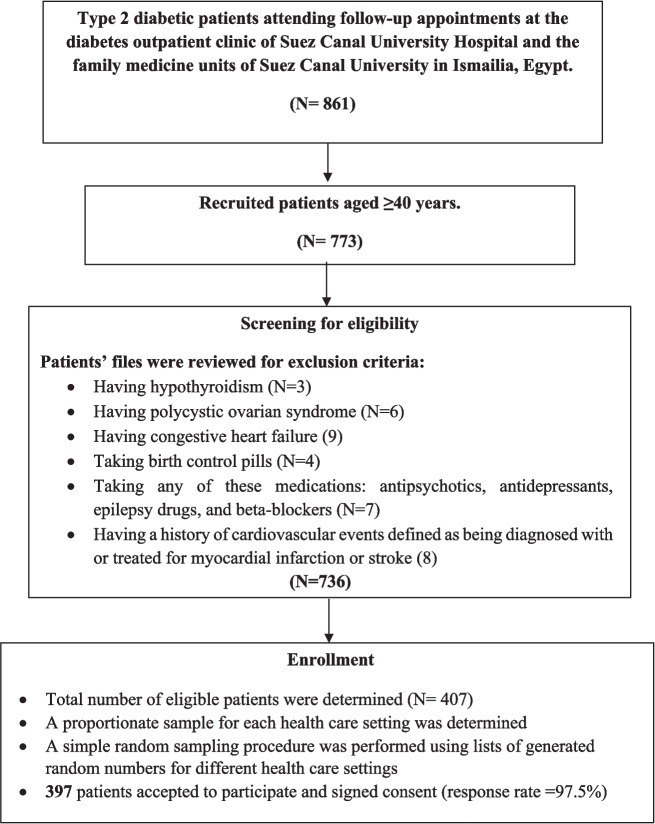
Flow diagram of patients’ enrollment

The sample size was calculated using G*Power 3.1.9.7 [22]. A minimum of 354 participants was required to achieve 80% power to detect an adjusted odds ratio (aOR) of at least 1.41 [23] for the association between visceral adiposity indices (X) and the high 10-year CVD risk (Y)—at a 95% level of confidence, Pr(Y=, X = 1) H0 of 0.52, R2 other X of 0.20, and a normal distribution of X (µ = 0, σ = 1). To account for a 15% non-response rate, the total sample was increased to 407 patients. The proportion of eligible patients in each setting was used to establish the sample size of participants. Participants were randomly selected from a list of coded-eligible participants’ files and invited to join the study using a random number generator of a web-based software tool (OpenEpi, available at https://www.openepi.com/Menu/OE_Menu.htm). The study’s objectives and methods were explained, and those who agreed provided written informed consent. A total of 397 patients participated, resulting in a 97.5% response rate (Fig. 1).

### Data collection tools

Participants’ self-reported data were collected through a structured face-to-face interview questionnaire administered by trained nurses during routine outpatient visits. The questionnaire captured demographic variables (age, gender, residency, education level, and family income), which have been previously established as factors influencing CVD risk [24–27]. It also included lifestyle-related data (physical activity and smoking status), which have likewise been identified as key contributors to CVD risk [28], along with medical history (hypertension, dyslipidemia, duration of diabetes, anti-diabetic, and anti-dyslipidemia medications) and family history (diabetes, myocardial infarction, or stroke). Physical activity level was assessed using a single-question approach with three response categories: inactive (no engagement in physical activity), lightly active (< 150 min of physical activity per week), and active (≥ 150 min of physical activity per week) [29]. Smoking status was categorized into a current smoker (smoked at least 100 cigarettes in their lifetime and currently smoking), non-smoker (never smoked), and former smoker (smoked at least 100 cigarettes in their lifetime but quit 1 year or more at the time of the interview) [30]. Hypertension and hyperlipidemia were defined based on participants’ self-reported medical history of a physician-diagnosed condition. The questionnaire was piloted on twenty participants not included in the study sample to ensure clarity and reliability. No modifications were required before the final implementation.

Participants’ clinical and anthropometric data were collected by trained nurses following standardized protocols. Measurements included blood pressure, weight, height, and WC. Blood pressure was measured twice on the left arm using a standard mercury sphygmomanometer after a 5-min rest, with the average reading recorded. Weight, height, and WC were taken with minimal clothing and no shoes. Weight and height were measured using the Beam Balance Scale with Height Rod, and WC was measured using a non-elastic flexible measuring tape. Weight was measured to the nearest 0.5 kg, height to the nearest centimeter, and WC to the nearest 0.5 centimeter (at the midpoint between the lowest ribs and iliac crest at the end of normal expiration). BMI was calculated based on weight (kg) divided by height (m^2^). Laboratory data were used to assess participants’ metabolic profiles, including fasting blood glucose (FBG) and lipid parameters [triglycerides (TG), total cholesterol (TC), high-density lipoprotein cholesterol (HDL-C), and low-density lipoprotein cholesterol (LDL-C)]. Blood samples were collected via venipuncture following an 8-h overnight fast. FBG was measured using the glucose oxidase-peroxidase (GOD-POD) enzymatic method, while lipid levels were determined using the enzymatic colorimetric method. Measurements were converted to standard units for compatibility with NVOI equations. Equations for the calculation of LAP, VAI, and METS-VF were as follows:

Equation (1): Calculation of LAP [31]:

1 $$\begin{array}{c}Male:\;LAP=\left(WC\left[cm\right]-65\right)\times\left(TG\left[mmol/L\right]\right)\\Females:\;LAP=\left(WC\left[cm\right]-58\right)\times\left(TC\left[mmol/L\right]\right)\end{array}$$

Equation (2): Calculation of VAI [14]:

2 $$\begin{array}{c}Males:\;VAI=\left\{WC\left[cm\right]\div\left[39.68+\left(1.88\times BMI\right)\right]\right\}\times\left(TG\left[mmol/L\right]\div1.03\right)\times\left(1.31\div HDL-C\left[mmol/L\right]\right)\\Females:\;VAI=\left\{WC\left[cm\right]\div\left[36.58+\left(1.89\times BMI\right)\right]\right\}\times\left(TG\left[mmol/L\right]\div0.81\right)\times\left(1.52\div HDL-C\left[mmol/L\right]\right)\end{array}$$

Equation (3): Calculation of METS-VF [8] (where $Ln=natural logarithm$):


3


METS-IR calculation [32]:

$$METS-IR=Ln\left[(2\times FBG\left[mg/dl\right]+TG\left[mg/dl\right]\right]\times \left[BMI\left[kg/{m}^{2}\right]\div Ln\left(HDL-C\left[mg/dl\right]\right)\right]$$

Participants’ 10-year CVD risk was determined using the WHO/ISH- 2019 CVD laboratory-based risk prediction chart for diabetics tailored for North Africa and the Middle East Region [6]. This chart assesses risk based on gender, age (≥ 40–79 years), smoking status, TC (mmol/l), and systolic blood pressure. The risk was categorized as low (< 10%), intermediate (10–< 20%), and high risk (≥ 20%) for fatal or non-fatal major cardiovascular events (myocardial infarction or stroke) in 10 years based on the World Health Organization guidelines.

### Statistical analysis

Given the non-normal distribution of continuous variables assessed by the Kolmogorov-Smirnov test, data were presented as medians and interquartile ranges (IQR). The Kruskal-Wallis test with Bonferroni correction was used for between-group comparisons and categorical variables were summarized as frequencies and percentages, with associations tested using Chi-square or Fisher’s exact test as appropriate. Binary logistic regression models evaluated associations between each visceral obesity index and the predicted high 10-year CVD risk (≥ 20%). Three models were constructed: a crude model, a model adjusted for socio-demographics and lifestyle factors (age, education, residence, income, and physical activity) (model 1), and a model further adjusted for diabetes duration, hypertension, taking anti-dyslipidemia medications, and blood lipids (TC, LDL-C, and TG) (model 2). Regression results models were presented as odds ratios (OR) with 95% confidence intervals (CI). The predictive performance of NVOIs for high CVD risk was assessed using receiver operating characteristic (ROC) curve analysis, with discriminative ability quantified using the C-statistic (area under the curve, AUC). Sensitivity and specificity were evaluated by determining the optimal cutoff values for each NVOI, based on the Youden index, which maximizes the sum of sensitivity and specificity. To compare predictive accuracy across groups, ROC curves were analyzed separately for males and females, and C-statistic (AUC) values were reported with 95% confidence intervals (CIs). AUC values indicate how well each index differentiates between individuals with and without high CVD risk, with a value of 0.5 representing no discrimination and 1.0 indicating perfect discrimination. Statistical comparisons between AUCs were performed using DeLong’s test, ensuring that performance differences between indices were statistically evaluated. All analyses were conducted using SPSS version 27.0, with statistical significance set at *p* < 0.05.

## Results

### Characteristics of study participants

The median age of study participants was 54 years (IQR = 15), and 52.6% were females. The median diabetes duration was 10 years (IQR = 10). Notably, 161 patients (40.5%) had a high 10-year CVD risk. Patients in the high CVD risk group were typically older, more likely to be male, urban residents, with higher income, lower physical activity, hypertension, dyslipidemia, longer diabetes duration, taking anti-diabetic and anti-dyslipidemic medications, and having higher systolic and diastolic blood pressure (*p* < 0.001). They also had higher BMI (*p* < 0.001), WC (*p* = 0.006 and* p* < 0.001 in males and females, respectively), worse FBG levels (*p* = 0.031), and lipid profile (*p* < 0.001) compared to those with low CVD risk (Table 1).

**Table 1:** General characteristics of patients with type 2 diabetes mellitus and its association with their 10-year cardiovascular disease risk, Ismailia, Egypt, 2023–2024 (*n* = 397)

**Characteristics**	**Total** (***n*** = **397)**	**10-year CVD risk**			***P*-value**
		Low *(n* = 67)	Moderate (*n* = 169)	High (*n* = 161)	
**Age (years), median (IQR)**	54.0 (15.0)	44.0 (5.0)	52.0 (9.0)	63.0 (7.0)	0.000 *
40–49	126 (31.7%)	56 (44.5%)	61 (48.4%)	9 (7.1%)	
50–59	131 (33.0%)	11 (8.4%)	92 (70.2%)	28 (21.4%)	0.000 *
60 +	140 (35.3%)	0	16 (11.4%)	124 (88.6%)	
**Sex, no. (%)**					
Males	188 (47.4%)	22 (11.7%)	69 (36.7%)	97 (51.6%)	0.000 *
Females	209 (52.6%)	45 (21.6%)	100 (47.8%)	64 (30.6%)	
**Residency**					
Urban	166(41.8%)	5 (3%)	43 (25.9%)	118 (71.1%)	
Suburban	138(34.8%)	24 (17.4%)	88 (63.8%)	26 (18.8%)	0.000 *
Rural	93 (23.4%)	38 (40.9%)	38 (40.9%)	17 (18.2%)	
**Education**					
Primary/preparatory	152 (38.3%)	21 (13.8%)	56 (36.8%)	75 (49.4%)	
Secondary/vocational	159 (40.1%)	31 (19.5%)	70 (44%)	58 (36.5%)	0.067
University/postgraduate	86 (21.6%)	15 (17.4%)	43 (50%)	28 (32.6%)	
**Family income, EGP**					
< 5000	109 (27.5%)	40 (36.7%)	50 (45.9%)	19 (17.4%)	
5000– < 10,000	147 (37.0%)	22 (15%)	69 (46.9%)	56 (38.1%)	0.000 *
≥ 10,000	141 (35.5%)	5 (3.5%)	50 (35.5%)	86 (61%)	
**Relevant family history**					
No	211 (53.1%)	35 (16.6%)	95 (45%)	81 (38.4%)	0.554
** Y**es	186 (46.9%)	32 (17.2%)	74 (39.8%)	80 (43%)	
**Cigarette smoking**					
Non-smokers	293 (73.8%)	58 (19.8%)	152 (51.9%)	83 (28.3%)	0.000 *
Current/former smokers	104 (26.2%)	9 (8.7%)	17 (16.3%)	78 (75%)	
**Physical activity**					
Inactive	243 (61.2%)	22 (9%)	101 (41.6%)	120 (49.4%)	0.000 *
Little active	97 (24.4%)	18 (18.6%)	47 (48.4%)	32 (33%)	
Active	57 (14.4%)	27 (47.4%)	21 (36.8%)	9 (15.8%)	
**Comorbidities**					
Hypertension	145 (36.5%)	7 (4.8%)	51 (35.2%)	87 (60%)	0.000 *
Dyslipidemia	150 (37.8%)	3 (2%)	55 (36.7%)	92 (61.3%)	0.000 *
** Duration of diabetes (years), median (IQR)**	10.0 (10.0)	5.0 (7.0)	8.00 (9.0)	13.0 (9.0)	0.000 *^a^
**Medications**					
Anti-diabetic	389 (98.0%)	62 (15.9%)	166 (42.7%)	161 (41.4%)	0.000 * ^F^
Anti-dyslipidemia	226 (56.9%)	29 (12.8%)	89 (39.4%)	108 (47.8%)	0.000 *
**Blood pressure (mmHg), median (IQR)**					
Systolic	130.0 (20.0)	110.0 (10.0)	130.0 (20.0)	140.0 (20.0)	0.000 *
Diastolic	80.0 (10.0)	80.0 (15.0)	80.0 (10.0)	85.0 (10.0)	0.000 *^b^
**BMI, median (IQR)**					
Males	29.62 (5.72)	26.63 (4.72)	28.28 (4.32)	31.77 (6.65)	0.000 * ^a^
Females	29.76 (8.66)	25.51 (2.68)	29.76 (4.60)	35.77 (7.80)	0.000 *
**WC, median (IQR)**					
Males	98.0 (11.50)	94.0 (10.0)	97.0 (9.0)	99.0 (11.0)	0.006 * ^a^
Females	90.0 (13.0)	84.0 (6.0)	90.0 (9.0)	98.0 (12.0)	0.000 *
**Metabolic profile (mmol/L), median (IQR)**					
TC	4.87 (1.79)	4.11 (0.7)	4.73 (1.55)	5.69 (1.92)	0.000*
HDL-C	1.1 (0.26)	1.2 (0.25)	1.11 (0.24)	0.98 (0.22)	0.000 *
LDL-C	2.57 (1.46)	2.18 (.86)	2.54 (1.55)	2.79 (1.61)	0.000 *
TG	2.25 (1.41)	1.64 (0.54)	2.03 (0.51)	3.54 (1.95)	0.000*
FBG	6.33 (2.5)	6.2 (1)	5.9 (1.3)	7.2 (4.5)	0.031*^c^

^*^Statistically significant at *p* < 0.05

^F^Fisher-Freeman-Halton exact test

^a^All pairwise comparisons were statistically significant except for low vs. moderate comparison (Bonferroni test)

^b^All pairwise comparisons were statistically significant except for moderate vs. high comparison (Bonferroni test)

^c^All pairwise comparisons were not statistically significant except for moderate vs. high comparison (Bonferroni test)

### Association of NVOI with 10-year CVD risk

Both sexes with high CVD risk showed significantly elevated LAP, VAI, and METS-VF levels compared to lower-risk groups (*p* < 0.001). Males exhibited higher LAP and METS-VF (87.55 and 7.31, respectively), while females had slightly higher VAI (3.43). Pairwise comparisons revealed varying NVOI patterns across risk categories, with some indices differentiating high-risk from lower-risk groups and others differentiating between specific pairs (Table 2). Regression analysis showed that VAI and METS-VF were independently associated with increased odds of high CVD risk in both sexes. Each unit increase in VAI raised the odds by 3.18 times (95% CI 1.61–6.26, *p* < 0.001) in males and 4.16 times (95% CI 1.26–13.68, *p* = 0.019) for females. Similarly, each unit increase in METS-VF was associated with a 7.39-fold increase in males (95% CI 1.03–52.85, *p* = 0.046) and a 7.8-fold increase in females (95% CI: 1.53–39.92, *p* = 0.014). LAP showed no significant association with high CVD risk (Table 3).

**Table 2:** Association of novel visceral obesity indices with 10-year cardiovascular disease risk among study participants (*n* = 397)

	**Total *N* = 397**	**10-year CVD risk**			***p*-value**
		Low(*n* = 67)	Moderate(*n* = 169)	High (*n* = 161)	
**Males** NVOI, median (IQR)					
LAP	87.55 (86.96)	51.92 (21.35)	68.51 (50.50)	133.32 (102.64)	0.000*^a^
VAI	3.21 (3.25)	2.05 (1.24)	2.64 (1.39)	5.09 (3.44)	0.000*^a^
METS-VF	7.31 (0.51)	6.91 (0.34)	7.18 (0.48)	7.46 (0.41)	0.000*
**Females** NVOI, median (IQR)					
LAP	67.0 (48.20)	43.90 (12.20)	66.55 (26.55)	110.0 (74.0)	0.000*
VAI	3.43 (1.98)	2.45 (0.46)	3.33 (1.17)	5.13 (2.30)	0.000*
METS-VF	6.76 (0.97)	6.07 (0.26)	6.71 (0.60)	7.25 (0.36)	0.000*

^*^Statistically significant at *p* < 0.05

^a^All pairwise comparisons were statistically significant except for low vs. moderate comparison (Bonferroni test)

**Table 3:** Novel visceral obesity indices as predictors of high 10-year cardiovascular disease risk (≥ 20%)

	**High 10-year CVD risk (≥ 20%** risk level**)**					
	Crude OR (95%CI)		Adjusted OR (95%CI)^a^			
			Model 1		Model 2	
**Males**						
LAP	1.03 (1.02–1.04)	0.000*	1.03 (1.01–1.05)	0.004*	1.03 (0.98–1.08)	0.253
VAI	2.19 (1.71–2.80)	0.000*	1.96 (1.22–3.16)	0.006*	3.18 (1.61–6.26)	0.000*
METS-VF	24.38 (8.26–71.94)	0.000*	26.07 (4.96–137.1)	0.000*	7.39 (1.03–52.85)	0.046*
**Females**						
LAP	1.04 (1.03–1.05)	0.000*	1.06 (1.04–1.09)	0.000*	1.04 (1.00–1.08)	0.067
VAI	3.20 (2.29–4.45)	0.000*	6.45 (3.01–13.82)	0.000*	4.16 (1.26–13.68)	0.019*
METS-VF	29.23 (10.58–80.77)	0.000*	22.70 (7.08–72.82)	0.000*	7.80 (1.53–39.92)	0.014*

^*^Statistically significant *p*-value at *p* < 0.05

^a^*Model 1* adjusted for sociodemographic and lifestyle factors (i.e., age, education, residence, income, and physical activity). *Model 2* adjustment for diabetes duration, hypertension, blood lipid (i.e., TC, LDL, and TG), and taking anti-dyslipidemia medications beside model 1 factors

### Performance of NVOI and optimal cut-offs for predicting high CVD risk

Novel visceral obesity indices demonstrated acceptable to excellent discriminative ability for high CVD risk, with AUC values ranging from 0.79 to 0.86 (*p* < 0.001). Discriminative ability was evaluated using ROC analysis, with sensitivity and specificity assessed at optimal cut-off values based on the Youden index, which maximizes the sum of sensitivity and specificity. Among males, VAI had the highest discriminative ability (AUC 0.83, 95% CI 0.77–0.89; *p* < 0.001), while in females, METS-VF demonstrated the highest performance (AUC 0.86, 95% CI 0.81–0.91; *p* < 0.001). While there were no statistically significant differences in discriminative ability among the indices in males, VAI showed a significantly higher C-statistic than LAP in females (mean difference: 0.028; *p* = 0.002). Males exhibited higher cut-off points for LAP and METS-VF, whereas females had a higher cut-off for VAI. Sensitivity was lower in males (63.92% to 74.23%) compared to females (76.56% to 87.5%). Among males, VAI showed the highest sensitivity (74.23%), while LAP had the highest specificity (94.51%). In females, METS-VF had the highest sensitivity (87.5%), while VAI demonstrated the highest specificity (82.76%). Sensitivity generally lagged behind specificity, except for METS-VF in females, where both measures were relatively balanced. Females had higher negative predictive values (NPVs) (88.9–93%), whereas males exhibited higher positive predictive values (PPVs) (77–92.5%). Likelihood ratios were higher in males (LR+ 3.14–11.63, LR− 0.3–0.4) than in females (LR+ 3.34–4.44, LR− 0.17–0.28) (Table 4).

**Table 4:** Performance of NVOI and optimal cut-offs for predicting high CVD risk

Indices	C-statistic (95% CI, *p*-value)	Pairwise differences (*P*-value)^a^		Cut-off value^b^	Sensitivity (95% CI)	Specificity (95% CI)	+ ve predictive value (95% CI)	− ve predictive value (95% CI)	+ ve likelihood ratio (95% CI)	− ve likelihood ratio (95% CI)
		*vs.* LAP	*vs.* VAI							
**Males**										
LAP	0.82 (0.76–0.88, 0.000*)			110.51	63.92 (53.5–73.4)	94.51 (87.6–98.2)	92.5 (83.9–96.7)	71.1 (65.2–76.3)	11.63 (4.90–27.63)	0.38 (0.29–0.50)
VAI	0.83 (0.77–0.89, 0.000*)	0.007 (0.601)		3.53	74.23 (64.3–82.6)	84.62 (75.5–91.3)	83.7 (75.8–89.4)	75.5 (68.5–81.4)	4.82 (2.94–7.92)	0.30 (0.21–0.43)
METS-VF	0.79 (0.73–0.86, 0.000*)	− 0.029 (0.244)	− 0.036 (0.217)	7.33	69.07 (58.9–78.1)	78.02 (68.1–86.0)	77.0 (69.0–83.5)	70.3 (63.3–76.5)	3.14 (2.09–4.73)	0.40 (0.29–0.54)
**Females**										
LAP	0.83 (0.76–0.89, 0.000*)			77.1	78.12 (66.0–87.5)	78.62 (71.0–85.0)	61.7 (53.5–69.3)	89.1 (83.6–92.9)	3.65 (2.61–5.12)	0.28 (0.17–0.45)
VAI	0.85 (0.80–0.91, 0.000*)	0.028 (0.002)*		4.08	76.56 (64.3–86.2)	82.76 (75.6–88.5)	66.2 (57.2–74.2)	88.9 (83.6–92.6)	4.44 (3.03–6.50)	0.28 (0.18–0.44)
METS-VF	0.86 (0.81–0.91, 0.000*)	0.035 (0.173)	0.008 (0.760)	6.83	87.50 (76.8–94.4)	73.79 (65.8–80.7)	59.6 (52.5–66.3)	93.0 (87.4–96.3)	3.34 (2.50–4.45)	0.17 (0.08–0.33)

^a^DeLong’s test

^b^Youden index J criterion

^*^Statistically significant *p*-value at *p* < 0.05

## Discussion

This study explores the relationship between three NVOIs (LAP, VAI, and METS-VF) and 10-year CVD risk in patients with T2DM. The significant association between LAP, VAI, and METS-VF and 10-year CVD risk supports our hypothesis. These NVOIs reflect the roles of demographic (sex, age), metabolic (FBG, TG, HDL-C), and anthropometric (BMI, WC, WhtR) factors, which had a significant association with CVD risk in univariate analysis. Limited research has explored the association between NVOI and CVD risk in T2DM patients, with most studies focusing on “CVD events” rather than risk prediction [19, 20]. While an initial study in China found no significant link between LAP, VAI, and CVD events in the diabetic population [19], a subsequent prospective study showed a stronger connection between these NVOIs and future CVD events [20]. Additionally, a recent cohort study conducted in China found a significant association between METS-VF and the increased risk of CVD events among populations with diabetes [18]. Our study, utilizing a predictive modeling approach, extends these findings by demonstrating a significant association between these indices and 10-year CVD risk in patients with T2DM.

Although our Bonferroni-corrected analysis revealed significant differences in LAP and VAI medians between the low and high and moderate and high CVD risk groups, no significant differences were observed between the low and moderate groups among males. A study by Zheng et al. [17] reported significant differences in LAP and VAI means between CVD risk groups using univariate analysis. However, the use of only two CVD risk categories and the absence of sex stratification in their study likely limited their ability to capture nuanced differences between groups. Additionally, the studies employed different CVD risk prediction tools, which assess risk based on different populations and risk factors, potentially influencing the classification and prediction of CVD risk and limiting direct comparisons between the two studies.

Multivariate analysis of our study results revealed that LAP had the lowest odds of high CVD risk and was not statistically significant in both sexes. In contrast, METS-VF and VAI showed higher odds of high CVD risk, with METS-VF demonstrating superior predictive ability, especially in females. A study on a Ghanaian population with T2DM [33] identified LAP as a significant predictor of 10-year CVD risk using the Framingham general cardiovascular risk profile. This discrepancy underscores the importance of considering CVD risk assessment tools and population-specific factors that could impact how TG and waist circumference (the components of LAP) relate to CVD risk.

Previous studies on general populations in Brazil [34] and the USA [35] identified VAI as a significant predictor of high CVD risk, which aligns with our findings. However, LAP’s role in predicting CVD risk remains inconsistent across studies potentially due to variations in population characteristics and metabolic profiles. The superior performance of VAI and METS-VF over LAP can be attributed to their inclusion of additional cardio-metabolic risk factors, such as HDL-C and FBG, which offer a more comprehensive evaluation of metabolic syndrome, a key risk factor for CVD [35]. Moreover, factors such as BMI, age, and WhtR, which are components of these indices, are independently recognized as risk factors for both metabolic syndrome and CVD [36–38]. Given the well-established association between metabolic syndrome and CVD [35], along with prior findings linking VAI and METS-VF to metabolic syndrome [8, 39], our results suggest that these indices may serve as effective predictors of CVD risk, partly through their association with metabolic dysfunction.

Unlike previous studies in the general population, where VAI showed a stronger association with CVD risk [17] and incidence of CVD events in males [40], our results indicate that diabetic females have a higher CVD risk per unit increase in VAI compared to males (4.16 vs. 3.18, respectively). This difference may be explained by males’ generally higher baseline CVD risk [41], reducing the additional impact of VAI. In contrast, females, with a typically lower baseline risk, experience a more pronounced increase in CVD risk with higher VAI. Differences in study populations may also contribute to the observed variation in sex-specific associations.

The superior predictive ability of METS-VF for CVD risk is likely due to its inclusion of METS-IR, which provides a comprehensive assessment of IR [32, 42] and metabolic syndrome [43, 44], both are key risk factors for CVD. Insulin resistance has been recognized as one of the most critical factors contributing to coronary artery disease [45]. No studies have examined sex differences in METS-VF’s ability to predict CVD risk in T2DM populations. A cohort study by Zhu et. al. [18] in China concluded that METS-VF could serve as a predictive index for CVD events, but sex-stratified analysis was not conducted, limiting comparison with our study. However, our results suggest that METS-VF's predictive power extends beyond IR and metabolic syndrome to encompass the impact of visceral fat accumulation on CVD risk.

Studies that examined the performance of NVOI in predicting high CVD risk in T2DM are limited. The C-statistic results in our study demonstrate the strong predictive value of these indices in identifying individuals at high CVD risk in both sexes (AUC = 0.79–0.866), highlighting their clinical usability [46]. In particular, the superior predictive performance of METS-VF in females may be attributed not only to its inclusion of METS-IR, which a recent Chinese study found to have the strongest predictive power for major adverse cardiac events in individuals with diabetes, outperforming other IR indices [47], but also to the stronger impact of insulin resistance (IR) on CVD risk in females. Previous research found a significant association between cytokines and METS-IR in females, with this effect being mediated by BMI [48]. Females in both studies had a higher BMI, likely amplifying the role of metabolic and inflammatory pathways in CVD risk. Despite lower METS-VF values in females, its stronger predictive power highlights the greater influence of IR and inflammation on cardiovascular risk in females.

VAI also demonstrated higher predictive performance than LAP in both sexes, with slightly better values in females. This contrasts with a Brazilian study that found LAP, not VAI, had significant predictive value for CVD risk using the Framingham risk score [49]. The differences between our study and the Brazilian study could be explained by variations in the study populations (less than 25% of participants in the Brazilian study had T2DM), the tools used for CVD risk assessment, and the lack of sex-stratified analysis in the Brazilian study, which may have missed important sex-specific differences. These findings, along with DeLong’s test, highlight the complexity of predicting CVD risk using LAP, VAI, and METS-VF, particularly regarding sex differences. The overlap in predictive components among the indices likely accounts for the similarities in performance across the indices, particularly in males and most comparisons in females. However, in females, VAI's performance was improved over LAP due to additional factors such as HDL-C and BMI**,** suggesting these indices may operate differently by sex.

In terms of cut-off points, the higher values for LAP and METS-VF in males and VAI in females reflect the sex-specific baseline differences. Females had higher sensitivity, meaning the indices were better at identifying high-risk females, while males showed higher specificity, making the indices more effective at excluding low-risk males. VAI showed the highest sensitivity in males, while METS-VF was most sensitive in females, further emphasizing sex differences in predicting high CVD risk. Youden’s index for LAP and VAI (0.56–0.58) in our study was higher than that reported in a healthy Iranian population [50], underscoring their greater predictive power in individuals with T2DM, likely due to their higher baseline risk. The indices showed higher negative predictive values (NPVs) in females (88.9–93%), meaning they are more effective at reassuring females when the test is negative. In contrast, higher positive predictive values (PPVs) in males (77–92.5%) provided stronger confirmation of high risk when the test was positive. Likelihood ratios (LR) also confirmed the indices’ effectiveness**,** with higher LR+ in males and lower LR− in females, reinforcing the importance of using sex-specific strategies when applying these indices for CVD risk prediction.

### Limitations

Although this study explored the association between NVOI indices and 10-year CVD risk, as well as their predictive performance in adults with T2DM, it has several limitations. First, its cross-sectional design prevents causal inferences between NVOI and CVD risk. Longitudinal studies are needed to evaluate these indices’ predictive ability over time. Second, the study was conducted in a single region of Egypt, limiting generalizability. Future research should include larger, multi-regional cohorts to enhance external validity. Third, the 2019 WHO/ISH risk prediction charts do not incorporate clinical event data, potentially reducing risk estimation precision. Investigating NVOIs alongside clinical event data could strengthen their predictive utility. Fourth, these charts exclude patients under 40 years old, limiting representation of younger individuals. Fifth, despite adjusting for major confounders, residual confounding from unmeasured factors such as dietary intake, stress levels, and genetic predisposition cannot be ruled out. Future studies should integrate more comprehensive lifestyle and genetic risk factors for a holistic understanding of CVD risk. Finally, reliance on self-reported data may have introduced recall and social desirability biases, affecting accuracy. While confidentiality measures helped mitigate these biases, future research should incorporate objective assessments for validation.

## Conclusions

Approximately 40% of our study participants were classified as having a high 10-year CVD risk. Both males and females with high CVD risk exhibited significantly higher levels of LAP, VAI, and METS-VF compared to those with low or moderate risk. VAI and METS-VF were independently and significantly associated with increased odds of high CVD risk in both sexes, whereas LAP showed no significant association. Notably, METS-VF demonstrated better discriminative ability for high CVD risk in females, while VAI performed better in males. Our findings suggest that VAI and METS-VF may serve as useful tools for CVD risk stratification in patients with T2DM patients, particularly in settings with limited resources. Clinicians may consider incorporating these indices into routine risk assessments to enhance early detection and prevention strategies.

## Data Availability

No datasets were generated or analysed during the current study.

## Abbreviations

CVD: Cardiovascular disease
T2DM: Type 2 diabetes mellitus
WHO/INH: World Health Organization/International Society of Hypertension
VAT: Visceral adipose tissue
WC: Waist circumference
WhtR: Waist height ratio
NVOI: Novel visceral obesity indices
LAP: Lipid accumulation product
VAI: Visceral adiposity index
METS-VF: Metabolic score of visceral fat
TG: Triglycerides
BMI: Body mass index
HDL-C: High density lipoproteins-cholesterol
FBG: Fasting blood glucose
IR: Insulin resistance
TC: Total cholesterol
LDL-C: Low density lipoproteins-cholesterol
IQR: Interquartile range
aOR: Adjusted odds ratio
CI: Confidence interval
ROC: Receiver operating characteristic
NPV: Negative predictive value
PPV: Positive predictive value
LR: Likelihood ratio
